# Soluble mediators derived from bronchial epithelium are able to drive Th2 differentiation in the context of rhinovirus infection

**DOI:** 10.1186/2045-7022-3-S1-O1

**Published:** 2013-05-03

**Authors:** Heidi Makrinioti, Ross Walton, Nikolaos Papadopoulos, Michael Edwards, Aurica Telcian, David Cousins, Kerstin Wanke, Luminita Stanciu, Mubeccel Akdis, Spyridon Megremis, Cezmi Akdis, Sebastian Johnston

**Affiliations:** 1 Airway Disease Infection Section, Imperial College London, UK; 2 University of Athens, Laboratory of Allergy and Clinical Immunology, Greece; 3 King’s College, Department of Immunobiology, UK; 4 Swiss Institute of Allergy and Asthma Research, Immunology Group, Switzerland

## Introduction

The majority of acute asthma exacerbations follow upper respiratory infections, and most are rhinovirus induced. The pathways by which a rhinovirus infection may lead to asthma development are still under scrutiny, but the role of bronchial epithelium in driving this mechanism has been considered of great importance. It has been shown that the epithelial derived cytokines IL25, IL33 and TSLP are upregulated in bronchial asthma and could be further induced by virus infection. We hypothesise that such cytokines might influence Th2 cell differentiation in the context of rhinovirus infection.

## Methods

Beas2B cells were infected with rhinovirus 1b (1MOI) or incubated with unifected Hela lysates and the supernatants were harvested 24 hours later. CD4+ T naive cells were isolated from CD Leukocyte Cone (buffy coat) and expanded for 12 days in the presence of anti-CD2, -CD3, -CD28 and IL2 stimulation. Th2 cells were also expanded out of the CD4+ T naive cells, in the presence of anti-CD2, -CD3, -CD28, -IL12 and IL4 stimulation. The already expanded Th0 cells were put into culture alone, in the presence of IL33, IL25 or TSLP (10 ng/ml) or with the supernatants from infected or uninfected cells above (4:1) for 12 more days. On Day 2, Day 6 and Day 12 of co culture intracellular levels of IFNγ, IL13, IL4 and FOXP3 were assessed by FACS.

## Results

On Day 12 of culture, IL13 levels were elevated in virus infected supernatant, IL33 and IL25 conditions at 10.5, 33.2, and 21.5 per cent in comparison to 2.45, 1.76 and 1.45 per cent on Th0 cells alone or in the presence of uninfected supernatants or TSLP. IL4 levels followed similar patterns at lower levels. The positive control Th2 cells express IL13 at 76.6 per cent and IL4 at 67.4 per cent. In the same conditions (virus, IL33 & IL25) trends for reductions in IFNγ positivity were observed, decreasing progressively from Day 2 through Day 6 to Day 12, while levels of expression of FOXP3 remained relatively constant. IL13, IFNγ and FOXP3 expression was not greatly different between the three timepoints in any other condition studied.

## Conclusion

Rhinovirus 1b infected bronchial epithelial cell supernatants, IL33 and IL25 promote Th0 cells to differentiate towards a Th2 phenotype and away from a Th1 phenotype during 12 days of co culture. This model of co culture might be helpful in investigating the role of the novel IL25, IL33 and TSLP in asthma development and exacerbation.

